# The Case for the Development of a Chagas Disease Vaccine: Why? How? When?

**DOI:** 10.3390/tropicalmed6010016

**Published:** 2021-01-26

**Authors:** Eric Dumonteil, Claudia Herrera

**Affiliations:** Department of Tropical Medicine, Vector-Borne and Infectious Disease Research Center, School of Public Health and Tropical Medicine, Tulane University, New Orleans, LA 70112, USA; cherrera@tulane.edu

**Keywords:** *Trypanosoma cruzi*, neglected tropical disease, parasite, Trypanosomiasis

## Abstract

Chagas disease is a major neglected tropical disease, transmitted predominantly by triatomine insect vectors, but also through congenital and oral routes. While endemic in the Americas, it has turned into a global disease. Because of the current drug treatment limitations, a vaccine would represent a major advancement for better control of the disease. Here, we review some of the rationale, advances, and challenges for the ongoing development of a vaccine against Chagas disease. Recent pre-clinical studies in murine models have further expanded (i) the range of vaccine platforms and formulations tested; (ii) our understanding of the immune correlates for protection; and (iii) the extent of vaccine effects on cardiac function, beyond survival and parasite burden. We further discuss outstanding issues and opportunities to move Chagas disease development forward in the near future.

## 1. Background

Chagas disease is a major neglected tropical disease, transmitted predominantly by triatomine insect vectors, but also through congenital and oral routes. It is endemic in the Americas, from southern Brazil and Chile to the US, but human migrations have turned it into a global disease with a significant number of cases in non-endemic regions such as Canada or Europe, among others [1,2]. There are at least 6 million cases in the Americas [3], but these estimates suffer from important uncertainties, as disease surveillance and reporting are highly heterogeneous among countries, and the disease burden could be higher. For example, recent estimates for Mexico, one of the most affected countries, range from less than 1 million [3] up to over 4 millions cases [4], and uncertainty is significant with potential publication bias [5].

Chagas disease control has so far relied on two main strategies—vector control and patient treatment. Vector control is mostly based on indoor spraying of residual insecticides to eliminate or at least reduce domestic triatomine populations inside dwellings, thereby reducing the incidence of new human cases. Despite some key achievements to control major vector species, and even eliminating transmission caused by *Triatoma infestans* and *Rhodnius prolixus* in some countries/regions [6,7], the continued presence of many other vector species able to transiently invade houses and maintain a low-level transmission implies a continued risk for human transmission and a challenge for effective vector control [8]. Therefore, there is growing recognition that a complete interruption of transmission to humans is not a feasible goal, and modeling suggests that vector control should be combined with other efforts to improve access to better care for patients in order to effectively reach the goals of the World Health Organization (WHO) 2020 London declaration, which call for a 100% certified interruption or control of Chagas disease [9].

The disease develops as an initial acute phase associated with a high parasitemia and non-specific signs of infection. Next, patients enter a chronic phase that is initially asymptomatic, with an apparent control of the parasitemia. However, 20−40% of patients will slowly develop clinical manifestations many years after the initial infection, the most common of which is chronic Chagasic cardiomyopathy (CCC). CCC is characterized by arrhythmias of increasing severity, leading to cardiac failure and death [1,10,11]. Other clinical manifestations include megaesophagus and megacolon, affecting about 10% of patients. The treatment of infected patients with trypanocidal drugs is being promoted to reduce morbidity and mortality associated with Chagas disease [10]. Benznidazole and nifurtimox are the two drugs of choice, and a 60-day treatment regimen is required for benznidazole, while a 60–90-day treatment regimen is required for nifurtimox. Such prolonged treatment courses present a logistic and economic burden in vulnerable populations where healthcare is limited. Both drugs are also associated with side effects that can be very severe, leading to frequent treatment interruptions [12,13]. Treatment is contraindicated in pregnancy and for patients with kidney or liver disease. Furthermore, treatment efficacy is questionable in the chronic stage of the disease, as it can reduce the blood parasite load but does not improve cardiac function [14,15,16]. Indeed, almost 20% of CCC patients will die within five years following their cardiac diagnosis, despite the efficacy of the benznidazole clearance of trypanosomes in the blood [15,16]. Thus, patient care is often only palliative, and significant mortality is observed [17,18,19].

## 2. The rationale for a Vaccine

Based on the above, new drugs and/or alternative strategies are still needed to improve the care of Chagasic patients, and a therapeutic vaccine would represent an attractive opportunity. An initial target product profile for such a therapeutic vaccine has been proposed [20]. It aims at preventing (desired target) or at least delaying (minimally acceptable target) the progression of CCC in patients with indeterminate Chagas disease (determined by antibody seropositivity), or in patients with early-stage evidence of clinical CCC (as determined by antibody seropositivity and cardiac clinical manifestations), to be used alone or in combination with drug therapy. An economic analysis of a therapeutic vaccine alone showed that it is highly cost-effective and frequently saves costs under a wide range of efficacy conditions by delaying CCC outcomes and side effects, and is also likely to provide a positive return on investment [21]. Furthermore, its combined use with current drugs could help bridging their toxicity gap, as it may allow for reducing drug doses and the associated severe side effects without compromising treatment efficacy. Indeed, modeling studies indicated that combining a therapeutic vaccine with a reduced dose drug treatment would result in more patients completing the treatment and would prevent more deaths than drug treatment alone [22]. Vaccines are economically dominant in a wide range of conditions, even when reducing the risk of disease progression as low as 5% [22]. Thus, it is expected that such a therapeutic vaccine would complement and help overcome the shortcomings of current vector control and drug treatments.

Furthermore, this initial indication may be expanded, as multiple additional uses of a vaccine have been proposed. Its potential use as a preventative vaccine is rather obvious and is supported by multiple pre-clinical studies (see below), although there are some concerns in terms of efficacy and cost-effectiveness that need to be considered. Nonetheless, alternative uses for a preventative vaccine may be for dogs, as these are considered to be a major domestic reservoir of the parasite, and they significantly increase the risk of human infection in many countries, as well as epidemiological conditions [23,24,25]. Thus, decreasing *T. cruzi* infection in dogs through vaccination may help reduce parasite domestic circulation. Another potential indication of a vaccine could be the prevention of congenital transmission [26]. Indeed, parasite transmission from an infected mother to her baby occurs in about 5% of pregnancies, and parasitemia is a key risk factor for the transmission of parasites [27,28]. Observational studies suggest that infected women treated at a young age do not transmit the parasite when pregnant later in life [29,30,31,32,33], which has led to the current recommendation of treating infected women of reproductive age [30,34]. Inducing a decrease in parasitemia with a therapeutic preconceptional vaccine in these women would thus be expected to reduce congenital transmission. Again, economic modeling confirmed that even a 25% efficacious vaccine would significantly reduce the number of congenital cases and would be cost-effective [35]. In addition, it may also provide an easy endpoint for the rapid clinical evaluation of vaccine efficacy, which could help accelerate vaccine development [26]. Thus, these additional indications for a Chagas disease vaccine further strengthen the rationale for its development.

## 3. Current Vaccine Platforms

An extensive variety of Chagas disease vaccine platforms have been tested in animal models over the years, ranging from live attenuated vaccines, DNA, recombinant virus or bacteria, and recombinant proteins, with a diverse range of formulations and adjuvants [36,37,38]. Initial studies served as a proof-of-concept to illustrate that controlling *T. cruzi* infection in mouse models is possible by inducing an immune response against parasite proteins. In the past few years, studies have further expanded (i) the range of platforms and formulations tested, (ii) our understanding of the immune correlates for protection, and (iii) the extent of vaccine effects beyond survival and parasite burden (Table 1). What emerges from these studies is that a substantial decrease in parasite burden (in the blood, cardiac, and skeletal muscle tissues) and improved survival can be achieved in mice through preventative vaccination with multiple vaccine platforms. A smaller number of studies have also shown a similar effect for the therapeutic administration of a vaccine in infected mice during the acute phase, as well as during the chronic phase. These results confirmed earlier proof-of-concept studies, but because of the diversity of methods and animal models, direct comparisons of immunogenicity and efficacy are not feasible. A notable new approach targets the immune response against an immunodominant α-Gal glycotope from *T. cruzi* mucin surface glycoproteins, which induce high antibody levels in Chagasic patients [39].

These studies have also confirmed the central role of IFNγ and the activation of CD4^+^ and CD8^+^ T cells in mediating parasite control, and a balanced Th1/Th2 response seems to provide a better outcome compared with a hyperpolarized response (Table 1). Additional specific subpopulations of immune cells are also emerging as complementary contributors that can mediate parasite elimination, including Th17 and NK cells, as well as trypanolytic/neutralizing antibodies [53,59,60,61,65]. While the confirmation of earlier results is encouraging, a major limitation of these approaches is that they remain limited in the breadth of the immune response assessed. Therefore, more integrative approaches from system immunology, such as those used for malaria, influenza, or yellow fever vaccines [70,71,72], may help reach a more comprehensive understanding of responses to vaccines and the correlates for protection against *T. cruzi*.

In terms of protection/prevention of tissue damage, several of these studies have shown that vaccination can reduce cardiac damage and dysfunction, in addition to parasite burden (Table 1). This is key because, as mentioned above, these are not necessarily correlated, as drug treatment administered during the chronic stage of the disease in humans can reduce the blood parasite burden, without improving cardiac function [14,15]. Delaying damage or improving cardiac function is indeed a central goal of vaccines. For example, an adenoviral vaccine expressing ASP-2 and TS [51], a DNA vaccine encoding TcG2 and TcG4 [40,41], or a recombinant Tc24 vaccine [48] can prevent the development of fibrosis when administered as therapeutic vaccines following infection. Similarly, the preventative vaccination with recombinant *Mycobacterium bovis* expressing trans-sialidase and cruzipain fragments [65], or with a DNA vaccine encoding cruzipain, Tc52, and Tc24 antigens [58], can prevent fibrosis and necrosis. A few studies have also shown improvements in cardiac function in response to vaccination, as assessed by electrocardiogram (EKG) [51,66,67]. It seems particularly remarkable that the therapeutic vaccination of mice with recombinant Adenovirus encoding ASP-2 and TS antigens during the chronic phase not only delayed the progression of cardiac damage and dysfunction, but even reversed these, as assessed by the extent of fibrosis and EKG alterations [51]. Thus, cardiac damage and CCC may be at least partially reversible, which provides strong support to further explore therapeutic vaccination against *T. cruzi* and its effects on cardiac function.

## 4. Challenges and the Way Forward

While the studies presented above are highly encouraging, some gaps in knowledge remain that need to be addressed. A first major limitation is that most studies have focused on assessing short term vaccine efficacy (acute phase), which is unlikely to be of relevant clinical use, and it is unclear how these results can translate into long term efficacy. A few studies of therapeutic vaccination administered during the chronic phase do support the conclusion that some of these vaccine formulations can control the parasite and prevent cardiac damage at this stage [48,51]. Similarly, preventative vaccination may provide long-term protection against infection [73]. Nonetheless, some vaccine effects may be transients, as observed following preventative vaccination with recombinant Adenovirus and MVA vectors encoding ASP-2 and TS, which decreased the parasite burden in the short term (acute phase), but not in the long term (chronic phase)[52]. In that respect, parasite tissue distribution remains a challenge, and studies using whole-body imaging in mice to detect fluorescent/bioluminescent parasites have been very valuable to address this point [52]. Thus, while measuring parasite burden in cardiac and skeletal muscle is key for vaccine efficacy, the assessment of additional tissues and longer follow-up times will be needed for a more reliable evaluation of vaccine efficacy. 

Parasite diversity represents another rarely addressed issue, although the potential lack of heterologous protection following vaccination has been demonstrated [74]. Indeed, *T. cruzi* presents extensive parasite diversity that is of a comparable magnitude to that observed among *Leishmania* species [75,76]. Thus, antigenic variation can be a significant issue for vaccine development. Epitopes from vaccine antigen TSA-1 were found to be conserved among parasite DTUs [77]. Similarly, analysis of Tc24 diversity among multiple strains from all DTUs indicated a high conservation and a strong purifying selection, which may limit antigen diversity [78]. Thus, these antigens may be effective against a wide diversity of strains, although this remains to be investigated. Nonetheless, another issue is that a large proportion of Chagasic patients harbor multiple parasite strains. This has been evidenced by changes in parasite genotypes following drug treatment, as well as direct genotyping [79,80,81]. While it is unclear if this represents sequential or simultaneous infections, interactions among parasite strains are likely to occur, as suggested by some co-infection studies in mice [82,83,84]. Further modelling suggests that co-infections in humans may result, in part, from insufficient immunity [85]. Thus, vaccine efficacy may be affected by co-infections, but this will be challenging to evaluate in a laboratory setting, given the limitations imposed by extrapolating from a necessary limited number of strain combinations. Field studies of natural infections should help assess this point. 

An additional challenge is that the large majority of vaccine studies described above focus on mouse models, and the extent to which the strong vaccine immunogenicity and efficacy observed can be extrapolated to humans remains unknown. Studies in dogs have shown promise in reducing the *T. cruzi* parasite burden [86,87,88,89,90], but their limited scope does not provide sufficient evidence supporting vaccine efficacy to delay or prevent cardiac dysfunction. Infectiousness of dogs may nonetheless be reduced by vaccination [91]. More recent studies have detected a recall cellular response by Tc24 and TSA1 vaccine antigens in Chagasic patients, indicating that they are processed during natural infection, supporting the potential use of these antigens in humans [92]. Furthermore, the first evaluation of this vaccine candidate in non-human primates indicated that it is safe, with no hepatic or renal alterations, and immunogenic, with humoral and cellular responses [93]. Thus, these encouraging results should spur additional studies to expand the work on murine models, and pave the way to clinical trials.

An additional aspect to be considered for developing a Chagas disease vaccine is the potential for scaling-up GMP production and regulatory issues of potential vaccine candidates and their further evaluation in clinical trials. So far, production processes for recombinant Tc24 and TSA-1 antigens are the only ones to undergo extensive process development and quality control. Specific mutations of cysteine residues were engineered in both antigens, to increase protein stability and yield, without compromising antigenicity, and scalable fermentation and purification steps have also been optimized and may be transferred for GMP production [45,46,94,95]. 

An overview of the current clinical development landscape for vaccines provides further insight on the potential of different platforms for further development (Table 2). Most vaccine platforms are amendable to large scale GMP production, although live attenuated and recombinant protein production may face variable hurdles, depending on specific organisms or antigens, respectively. In the case of *T. cruzi*, the large-scale production, storage, and distribution of a potential live attenuated vaccine would certainly be most challenging because of the multiple constraints of cultivating a eukaryotic parasite under GMP guidelines and consistently maintaining its viability. Additional regulatory limitations may be faced for clinical trials. While multiple vaccine platforms against infectious diseases are undergoing clinical development in Phase 1 studies, only live attenuated and recombinant protein vaccines are readily progressing to Phase 2 and Phase 3 studies (Table 2). An important concern of DNA vaccines for example is their limited immunogenicity in humans, although multiple strategies are being investigated to boost their efficacy [96]. On the other hand, Adenovirus and other viral-based vaccines may face safety issues, and pre-existing immune cross-reactivity against the virus vector may interfere with vaccination [97,98]. Based on these data, a Chagas disease vaccine based on recombinant proteins may represent the quickest path toward clinical trials, and Tc24 and TSA-1 antigens are well poised for such a development.

In conclusion, recent advances have confirmed the potential of vaccines against Chagas disease and have solved some of the key challenges. A remaining challenge is the political will and investment needed to move a vaccine into clinical development for a neglected tropical disease such as Chagas disease. Indeed, despite its large health burden [99], it remains one of the most neglected of the neglected diseases, and further steps will require bold decisions from multiple stakeholders and partners to move this vaccine candidate into clinical trials [100,101,102].

## Figures and Tables

**Table 1:** Recent Chagas Disease Vaccine Platforms and Formulations.

	Antigens and Use	Adjuvants and Delivery Systems	Immune Response	Efficacy Against Parasite	Efficacy Against Cardiac Damage and Dysfunction	References
**Therapeutic vaccines**						
**DNA**	TcG2+TcG4, therapeutic during acute phase	Plasmids or nano plasmids	CD4^+^ and CD8^+^ producing IFNg, PRF, and GRZ	Decreased parasite burden in cardiac and skeletal muscle	Decrease in fibrosis in heart and skeletal muscle, decrease in oxidative stress	[40,41]
**DNA**	Cruzipain and Chagasin plasmids with *Salmonella* carrier, therapeutic during acute phase	GM-CSF expression plasmid	Increased IFNγ and antibodies	Decreased parasite burden in blood and heart, and increased survival	Decreased cardiac inflammation	[42]
**Peptides**	10 peptide epitopes mixture, therapeutic during acute phase	TLR4 agonist (MPLA)	Increased IFNγ	Decreased cardiac parasite burden, increased survival	N/A	[43]
**Recombinant proteins**	Tc24, TSA-1 and their optimized variants, therapeutic during acute phase	TLR4 agonists (E6020, MPLA, GLA), TLR9 agonist (CpG), nanoparticles	Antibodies, IFNγ, and CD4^+^ and CD8^+^ activation	Decreased cardiac parasite burden, increased survival	Decrease in cardiac inflammation and fibrosis	[44,45,46,47]
**Recombinant proteins**	Tc24, therapeutic during chronic phase	TLR4 agonists (E6020)	High IFNγ and low IL4, and antibodies	Decreased parasitemia	Decrease in cardiac inflammation and fibrosis	[48]
**Recombinant proteins**	Tc24-C4, therapeutic combined with low dose Benznidazole	TLR4 agonist (E6020)	Increased IFNγ, IL12, TNFa, IL2, IL4 and IL10, and CD4^+^ and CD8^+^ T cell activation	Decreased parasitemia, increased survival	N/A	[49]
**Viral vectors**	Recombinant Adenovirus expression ASP2, therapeutic during acute phase		TNFa, iNOS, TLR4, and IL-10 expression in the liver	Increased survival, decreased parasite burden in liver	N/A	[50]
**Viral vectors**	Recombinant adenovirus expressing ASP2 and TS, therapeutic during chronic phase		IFNγ and CD8^+^ T cells	Increased survival	Decreased cardiac fibrosis and dysfunction	[51]
**Preventative vaccines**						
**Live parasites**	Drug-cured primary infection, preventative	N/A	N/A	Sustained decrease in parasite burden in all body	N/A	[52]
**Live parasites**	Live attenuated parasite (inducible expression of alpha-toxin, and cecropin A), preventative	N/A	IFNγ, TNFa, CD4^+^ and CD8^+^ T cell activation, antibodies, and NK cells	No detectable parasites	Decrease in cardiac inflammation	[53,54]
**Live parasites**	Live attenuated parasite (TCC attenuated strain), preventative short term	IFNγ expressing plasmid	Antibodies and mixed Th1/Th2 response	Decreased parasitemia and increased survival	N/A	[55]
**DNA**	TcG2+TcG4, preventative short term	Plasmids alone or with *Trypanosoma rangeli* and/or Quil A as adjuvants	Antibodies, CD4^+^ and CD8^+^ producing IFNγ, TNFa, and PRF	Decreased parasite burden in cardiac and skeletal muscle		[56]
**DNA**	Cruzipain plasmid, preventative short term	GM-CSF plasmid	Antibodies and DTH	Decreased parasitemia, increased survival	Decreased cardiac tissue damage	[57]
**DNA**	Cruzipain, Tc52, Tc24 plasmids, preventative short term	*Salmonella enterica* carrier	Trypanolytic antibodies, DTH, IFNγ, IL12, and IL10	Decreased parasitemia, increased survival	Decreased cardiac tissue inflammation, necrosis	[58]
**Recombinant proteins**	Cruzipain fused with staphylococcal superantigen, preventative short term	TLR9 agonist (CpG)	Neutralizing antibodies and DTH	Decreased parasitemia and increased survival	N/A	[59]
**Recombinant proteins**	Recombinant Traspain, Cruzipain and ASP-2 fusion protein, preventative short term	c-di-AMP adjuvant (STING agonist)	Neutralizing antibodies, CD4^+^ and CD8^+^ T cell activation, IFNγ, TNFa, IL2, and IL17	Decreased parasitemia and increased survival	Decreased cardiac damage (CK, CK-MB), decreased necrosis and inflammation in the heart and skeletal muscle	[60]
**Recombinant proteins**	Recombinant Tc52 fragment, preventative short term	c-di-AMP adjuvant (STING agonist)	Antibodies, CD4^+^ and CD8^+^ T cell activation, IFNγ, and IL17	Decreased parasitemia and increased survival	N/A	[61]
**Recombinant proteins**	TcTASV, preventative short term	Unlipidated Outer Membrane Protein 19 of *Brucella abortus* (U-Omp19) as adjuvant	Trypanolytic antibodies, IFNγ, and IL17	Decreased parasitemia and increased survival	N/A	[59]
**Recombinant proteins**	Enolase, preventative short term	Freund complete/incomplete adjuvant	Antibodies, IFNγ, and IL2	Decreased parasitemia and increased survival	Decreased cardiac and skeletal muscle inflammation	[62]
**Recombinant proteins**	Trans-Sialidase fragment, Preventative short term	ISPA lipidic cages, ISCOMATRIX, or Freund adjuvant	Trypanolytic antibodies, IFNγ, CD4^+^ and CD8^+^ T cell activation, Treg activation	Decreased cardiac parasite burden, increased survival	Decreased cardiac inflammation	[63,64]
**Glycotope**	αGal glycotope, preventative short term	TLR4 agonist (Liposomal-monophosphoryl lipid A)	Trypanolytic antibodies, CD4^+^ and CD8^+^ T cell activation	Decreased parasite burden in multiple tissues, increased survival	Decreased cardiac inflammation and necrosis	[39]
**Viral vectors**	Recombinant Adenovirus and modified Vaccinia Ankara virus expressing ASP-2 and Trans-sialidase, preventative vaccination	PBS		Decreased parasite burden during the acute phase in all body, but no impact on long-term burden during chronic phase	N/A	[52]
**Bacterial vectors**	Recombinant *Mycobacterium bovis* (BCG) expressing trans-sialidase and cruzipain fragments, preventative short term		Trypanolytic antibodies, and DTH, CD4^+^ expressing IFNγ, IL17, IL10, and CD8^+^	Increased survival	Decreased cardiac inflammation and fibrosis	[65]
**Heterologous prime-boost combination**	*Salmonella enterica* expressing Traspain and ASP-2	TLR9 agonist (CpG)	Increased IFNγ, IL17, low IL4, CD4^+^, and CD8^+^ T cell activation	Decreased parasite burden in blood, heart and skeletal muscle, and increased survival	Decreased inflammation and improved EKG	[66]
**Heterologous prime-boost combination**	Recombinant 80 kDa prolyl oligopeptidase (Tc80) and plasmid DNA	TLR9 agonist (CpG)	Increased IFNγ, IL2, TNFa, CD4^+^, and CD8^+^ T cell activation	Decreased parasitemia, and increased survival	Decreased cardiac inflammation, damage (CK and CK-MB), improved EKG	[67]
**Heterologous prime-boost combination**	Recombinant Tc52 and plasmid DNA with Salmonella carrier, preventative short term	TLR9 agonist (CpG)	Trypanoplytic antibodies, Increased IFNγ, IL10, CD4^+^, and CD8^+^ T cell activation	Decreased parasitemia, and increased survival	Decreased cardiac inflammation	[68]
**Heterologous prime-boost combination**	TcG1, TcG2, and TcG4 expression plasmids and recombinant proteins, preventative long term	IL2 and GM-CSF plasmids	Increased IFNγ, TNFa, CD4^+^ and CD8^+^ T cell activation	N/A	N/A	[69]

PRF—perforin; GZN—granzyme; DTH—delated type hypersensitivity; TLR—Toll-like receptor; IL—interleukin; IFN—interferon; TNF—tumor necrosis factor; EKG—electrocardiogram; N/A—not applicable.

**Table 2:** Production and Clinical Development of Vaccines against Infectious Diseases.

Vaccine Type	Ease of Production	Clinical Development *	Potential Issues
**Attenuated**	Variable	140 Phase 1 71 Phase 2 43 Phase 3	Reversal of attenuation, storage, and distribution of a live vaccine
**DNA**	++++	207 Phase 1 61 Phase 2 0 Phase 3	Limited immunogenicity in humans
**Adenovirus**	+++	69 Phase 1 21 Phase 2 1 Phase 3	Risk of adverse effects and immunity to vector
**Recombinant proteins**	++++ or variable	195 Phase 1 76 Phase 2 55 Phase 3	Most widely accepted, safe and immunogenic

* Number of vaccine studies of the different phases registered in ClinicalTrials.gov, excluding COVID-19 vaccines (as of 30 October 2020).

## Data Availability

Not applicable.
